# Generation of TALEN-mediated FH knockout rat model

**DOI:** 10.18632/oncotarget.11429

**Published:** 2016-08-19

**Authors:** Dandan Yu, Yali Zhong, Xiaoran Li, Yaqing Li, Xiaoli Li, Jing Cao, Zhirui Fan, Huijie Fan, Long Yuan, Benling Xu, Yuan Yuan, Hongquan Zhang, Zhenyu Ji, Jian-Guo Wen, Mingzhi Zhang, Jahn M. Nesland, Zhenhe Suo

**Affiliations:** ^1^ Department of Oncology, The First Affiliated Hospital of Zhengzhou University, Zhengzhou, Henan, China; ^2^ Departments of Pathology, The Third Affiliated Hospital of Zhengzhou University, Zhengzhou, Henan, China; ^3^ Department of Surgery, The Affiliated Cancer Hospital of Zhengzhou University, Zhengzhou, Henan, China; ^4^ Department of Cancer Biotherapy, The Affiliated Cancer Hospital of Zhengzhou University, Zhengzhou, Henan, China; ^5^ Department of Pathology, Capital Medical University, Beijing, Fengtai, China; ^6^ Department of Anatomy, Histology and Embryology, Peking University Health Science Center, Beijing, Fengtai, China; ^7^ Department of Oncology, Henan Academy of Medical and Pharmaceutical Sciences, Zhengzhou University, Zhengzhou, Henan, China; ^8^ Institute of Clinical Medicine, The First Affiliated Hospital of Zhengzhou University, Zhengzhou University, Zhengzhou, Henan, China; ^9^ Department of Pathology, The Norwegian Radium Hospital, Oslo University Hospital, Oslo, Norway; ^10^ Department of Pathology, Institute of Clinical Medicine, Faculty of Medicine, University of Oslo, Oslo, Norway

**Keywords:** TALEN, fumarate hydratase, gene technology, obesity, heterozygote

## Abstract

Transcription activator-like effector nucleases (TALENs) are valuable tools for precise genome engineering of laboratory animals. Here we utilized this technique for efficient site-specific gene modification to create a fumarate hydratase (FH) gene knockout rat model, in which there was an 11 base-pair deletion in the first exon of the FH gene in 111 rats. 18 live-born targeted mutation offsprings were produced from 80 injected zygotes with 22.5% efficiency, indicating high TALEN knockout success in rat zygots. Only heterozygous deletion was observed in the offsprings. Sixteen pairs of heterozygous FH knockout (FH+/−) rats were arranged for mating experiments for six months without any homozygous KO rat identified. Sequencing from the pregnant rats embryo samples showed no homozygous FH KO, indicating that homozygous FH KO is embryonically lethal. Comparatively, the litter size was decreased in both male and female FH+/− KO rats. There was no behaviour difference between the FH+/− KO and the control rats except that the FH+/− KO male rats showed significantly higher body weight in the 16-week observation period. Clinical haematology and biochemical examinations showed hematopoietic and kidney dysfunction in the FH+/− KO rats. Small foci of anaplastic lesions of tubular epithelial cells around glomeruli were identified in the FH+/− kidney, and these anaplastic cells were comparatively positive for Ki67, p53 and Sox9, and such findings are most probably related to the kidney dysfunction reflected by the biochemical examinations of the rats. In conclusion, we have successfully established an FH+/− KO rat model, which will be useful for further functional FH studies.

## INTRODUCTION

Recently, with the development of genetic engineering technology, rapidly increasing researches focus on gene edited rats as an optimal model for human disease studies, including cancer, because rats are physiologically more similar to human than mice [1–3]. However, because of the high cost and technical demanding, production of genetically engineered rats has been severely hampered. Fortunately, several promising novel methods of efficient, targeted genome editing technology have emerged, including zinc finger nucleases (ZFN) [4], transcription activator-like effector nucleases (TALENs) [5] and the recently developed clustered regularly interspaced short palindromic repeat (CRISPR) [6], all useful to efficiently modify the genome DNA sequence. TALENs are customizable, DNA binding nucleases that can be targeted to bind almost any sequence in the genome. Every TALEN is consisted of two domains, one is composed by repeating units to form DNA binding domain, and the other is FoK I endonuclease, which has the unspecificity activity of endonuclease. Each repeating unit is composed by 33–35 amino acids, and the adjacent amino acids of 12 and 13 can specifically recognize DNA nucleotide sequences, such as the amino acids of NI, NN, NG, HD can identification A, G, T, C bases, respectively. Therefore, TALEN can edit the genome with higher accuracy and specificity, which can be easily designed, cloned, assembled and tested in a molecular biology laboratory.

In this study, we utilized the TALENs for efficient site-specific gene modification to create a novel gene-fumarate hydratase (FH) knockout (KO) rat model. FH plays an important role in the Krebs tricarboxylic acid (TCA) cycle, which catalyses the hydration of fumarate into malate. Heterozygous mutations of FH are responsible for atypical uterine leiomyomas [7], hereditary leiomyomatosis [8] and renal cell cancer [7, 9]. Moreover, it was shown that FH deficiency could lead to metabolic disorder with severe encephalopathy, seizures, and poor neurological outcomes [10]. Dysfunctional FH in cells and tissues attributes to accumulating high levels of fumarate, which has been proposed to promote cancer development, indicating a tumor suppressor of FH [7]. The purposes of our current study are to explore whether germ-line FH KO rats could be generated and to know the general influence of the FH KO in the rat model.

## RESULTS

### TALEN design and assembly

**Figure 1:**
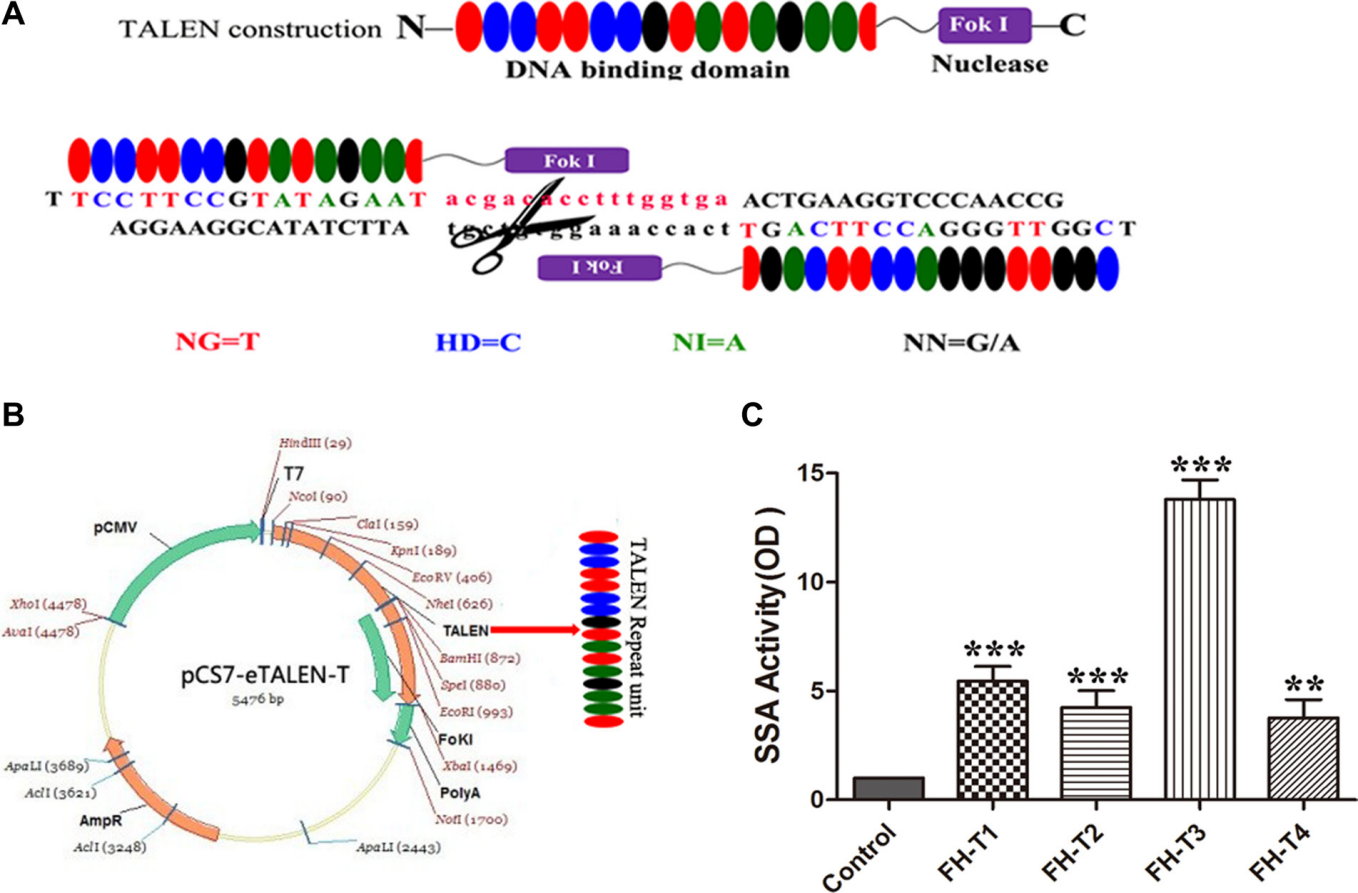
TALEN design and assembly (**A**) shows the diagram of TALEN. (**B**) shows the assembly of TALEN. (**C**) shows the efficiency detect for the TALEN plasmids. The results shown that the FH-T3 has the most efficiency for knockout. *n* = 3. *P < 0.05, **P < 0.01, ***P < 0.001.

TALEN plasmids targeting FH gene by using the Golden gate method were constructed and illustrated graphically in Figure 1A and 1B. All TALENs targeted the rat FH gene, in which the repeat unit of TAL specifically binds the DNA sequences and the pair of Fok I endonuclease precisely knockouts the FH sequences. Four pairs of TALEN plasmids targeting on the exon 1 of FH gene are shown in Table 1. The most effective plasmid was screened, showing that the FH-T3 has the most effective activity (Figure 1C).

**Table 1:** OGDH target sequences

Name		Target Sequences (5′ to 3′)
FH-T1	Sense strand	F- TCGTTCCCGCGGGTC
	Antisense strand	R- AGCAAGGGCGCCCAG
FH-T2	Sense strand	F- GGCCACCCTCCCACGGTGT
	Antisense strand	R-CCGGTGGGAGGGTGCCACA
FH-T3	Sense strand	F- ACGACACCTTTGGTGA
	Antisense strand	R-TGCTGTGGAAACCACT
FH-T4	Sense strand	F- CCAACCGATAAGTATT
	Antisense strand	R-GGTTGGCTATTCATAA

### TALEN efficiency and TALEN-targeted product sequencing

**Figure 2:**
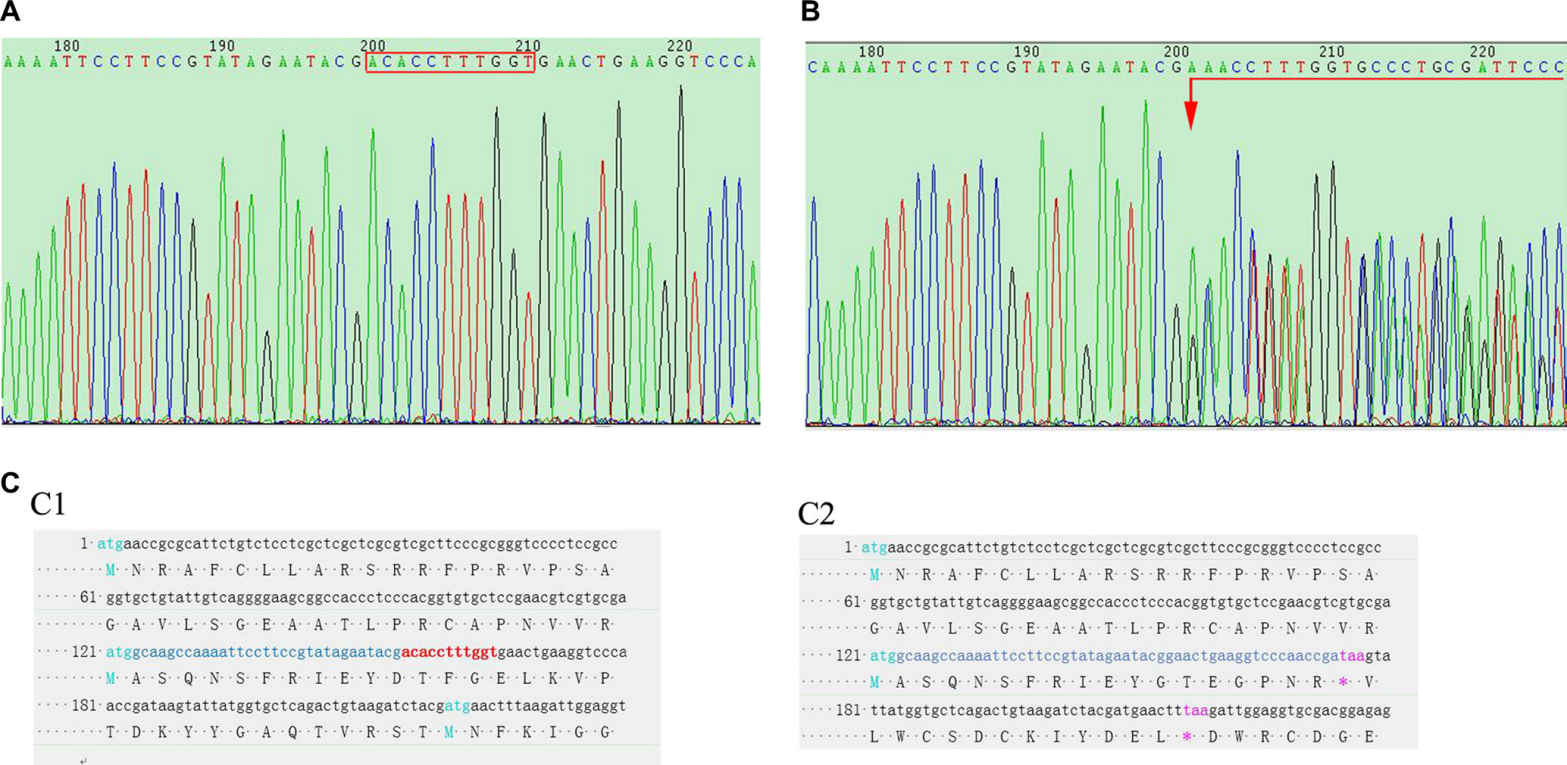
Rat tail DNA sequencing (**A**) shows FH gene sequencing result of a WT rat, where the bases in the red rectangle are the TALEN targets. (**B**) shows FH sequencing result in a FH+/− KO rat, on which, chaos sequencing waives begin from the first TALEN targeted base (arrow head). (**C**) sequencing results of the FH exon 1 cDNA in the WT and FH+/− KO rats, where the red bases in C1 shows the TALEN targets in WT rat, and these bases are missing in the FH+/− KO rat on C2 with a terminator “taa” 20 bases after the TALEN targets, which results in a very short transcript.

TALEN mRNAs were injected into the cytoplasm of 1-cell SD rat embryos *in vitro* and then the embryos were transferred to pseudopregnant recipients. In total, 18 live-born targeted mutation offsprings were produced from 80 injected zygotes. The offsprings were then sequencingly screened for targeted disruption of FH exon 1 as illustrated in Figure 2B. The wild type (WT) sequence was found identical to that in the rat genome database (Figure 2A). Compared to the sequence in the WT rats, heterozygous rat founders were identified with 11 base deletions in one allele in exon 1. The mutation is a non-triple base deletion as shown in Figure 2C1, marked in red, and an early terminator “taa” was created in the exon 1 transcript as shown in Figure 2C2 marked in purple, which resulted in a very short transcript.

### Litter size observation and embryos sequencing

**Figure 3:**
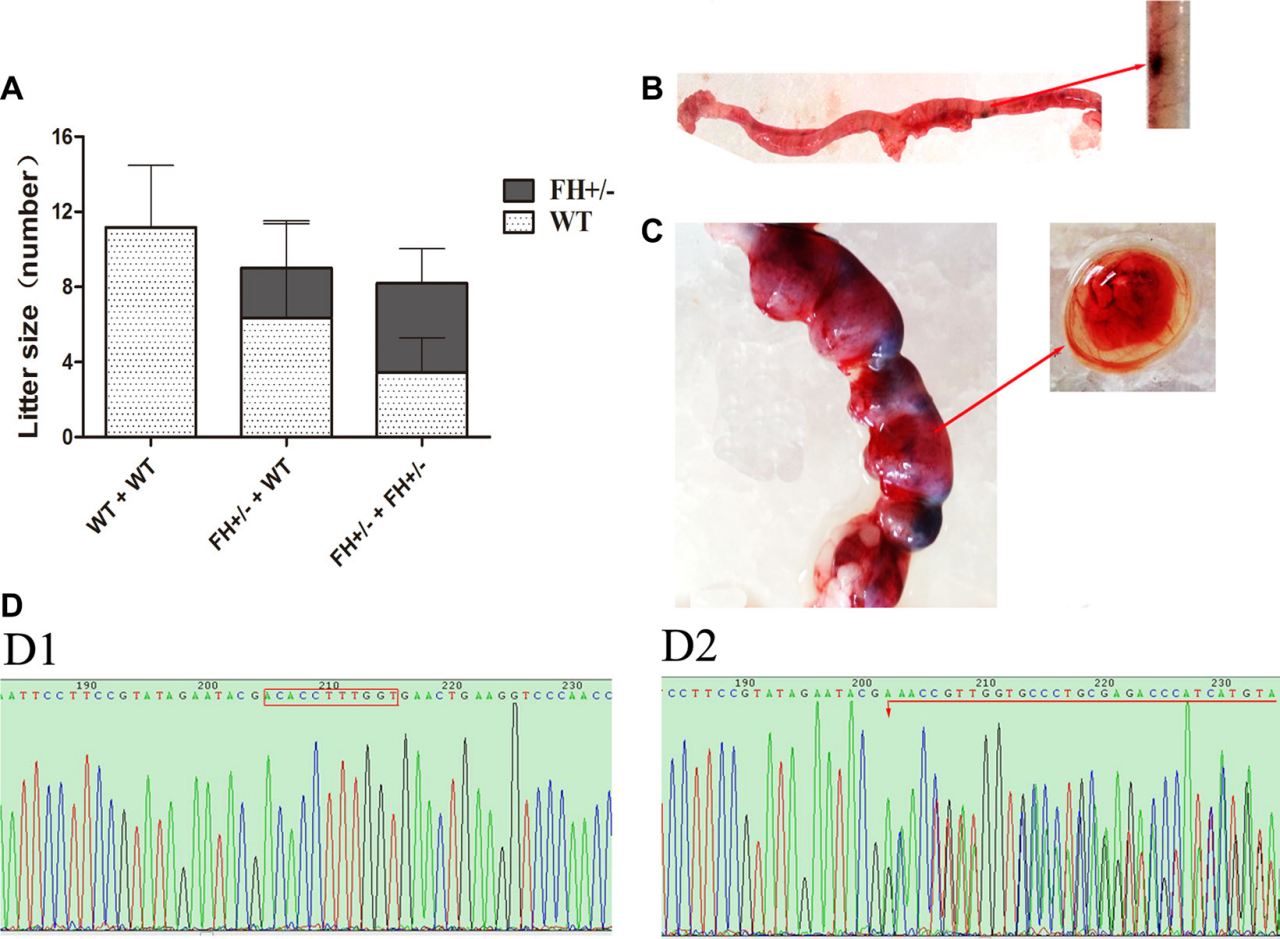
Litter size study and rat embryo sequencing (**A**) shows the results of litter size observation in histograms. As shown on the histograms, the WT rat -WT rat mating groups show about 11 new born rats per litter, while the WT rat- FH+/−KO rat mating groups show 9 per litter with 29.6% decrease; and the FH+/− KO rat-FH+/− KO rat mating groups show 8 per litter with 58.0% decrease. (**B**) shows embryos from an E8.0 rat. (**C**) shows embryos from an E15.0 rat. **D1** and **D2** show the results of FH sequencing in WT and FH+/− KO embryos, respectively.

To explore whether FH+/− influenced litter size, the following mating experiments were performed: female founder rats mated with WT SD males, and heterozygous offsprings mated with heterozygous offsprings. Interestingly, compared with the reproduction capability shown by WT/WT mating, the litter size was declined with 27.4% when heterozygous KO rats mated with WT rats, and declined with up to 40.0% when heterozygous KO rats mated to heterozygous KO rats as shown in Figure 3A. The litter size of the heterozygous FH KO (FH+/−) rat was increased in the heterozygous mating groups than in the heterozygous with WT mating groups, with a rate of 58.0%, compared to the rate of 29.6%. However, no homozygous FH KO rats were found in a total of 16 pairs of the heterozygous KO and heterozygous KO mating rats within the six month experiment. In order to further clarify embryo lethality of homozygous FH KO, embryos from E8.0 (Figure 3B) and E15.0 gestation rats (Figure 3C) were obtained. PCR products from these embryos were applied for DNA sequence analysis as illustrated in Figure 3D1 (WT) and Figure 3D2 (FH+/−). Again, no homozygous FH KO rat was revealed in these embryos, indicating FH–/− KO embryo lethality.

### FH+/− KO resulted in reduced FH gene and protein expression

**Figure 4:**
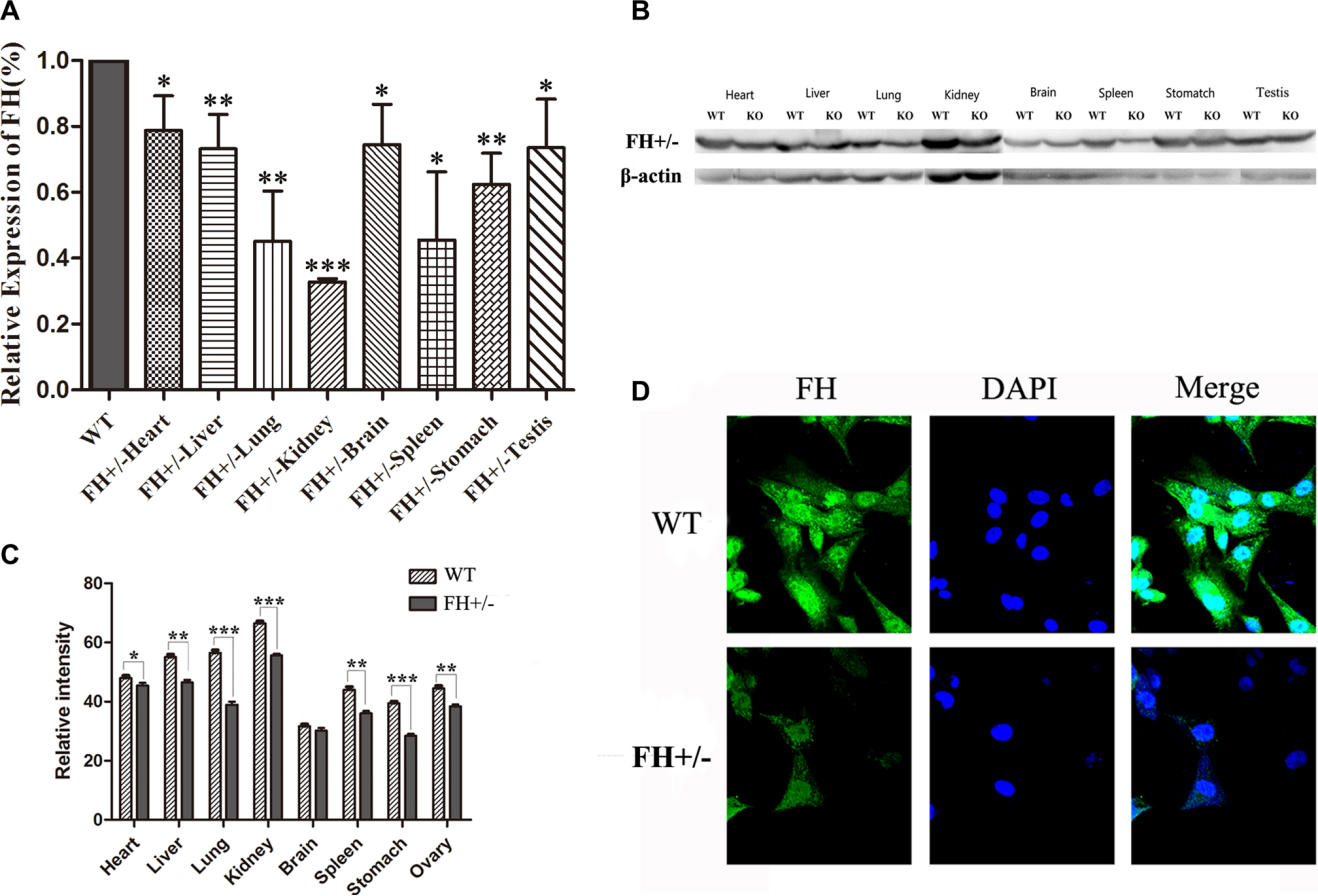
FH mRNA and protein expression examination Results of the FH mRNA detected by RT-qPCR and protein expression examined with Western blotting in the heart, liver, lung, kidney, brain, spleen, stomach and testis tissues of WT and with FH+/− KO rats are shown on (**A**) and (**B**), respectively. (**C**) shows the corresponding histograms of protein expression in the tissues from 3 WT rats and 3 FH+/− KO rats. Representative immunofluorescence microscopy of the FH protein in the primary lung fibroblast cells from WT and FH+/− KO rats. Cells are stained with DAPI to visualize the nuclei (blue). FH (green) is localized in the cytoplasm. All photographs were originally taken at 200× (**D**). * means *P* < 0.05, and ** means *P* < 0.01. *n* = 3.

To further analyze the FH+/− status in the rats, the expression of FH gene and protein was analyzed with RT-qPCR and Western blotting, respectively. Compared with the WT rats, the expression of FH gene was variably declined in the tissues of the heart, liver, lung, kidney, brain, spleen, stomach and testis as shown in Figure 4A, with a reduce rate of up to 67.2% in kidney (*P* < 0.001). Similarly, the expression of FH protein in the FH+/− KO rats was also variably reduced in the tissues of the heart, liver, lung, kidney, brain, spleen, stomach and testis as shown in Figure 4B, and the histograms of the protein expression are shown on Figure 4C, with a reduction rate of up to 16.3% in the kidney (*P* < 0.001). Expression of FH protein was also examined with an immunofluorescence microscope in the primary fibroblasts isolated from the FH+/− KO rats and control rats. As shown in Figure 4D, cytoplasmic FH protein expression is detected in the fibroblasts of the control rat, but reduced immunostaining was revealed in the fibroblasts of the FH+/− KO rat.

### Behaviour observation

**Figure 5:**
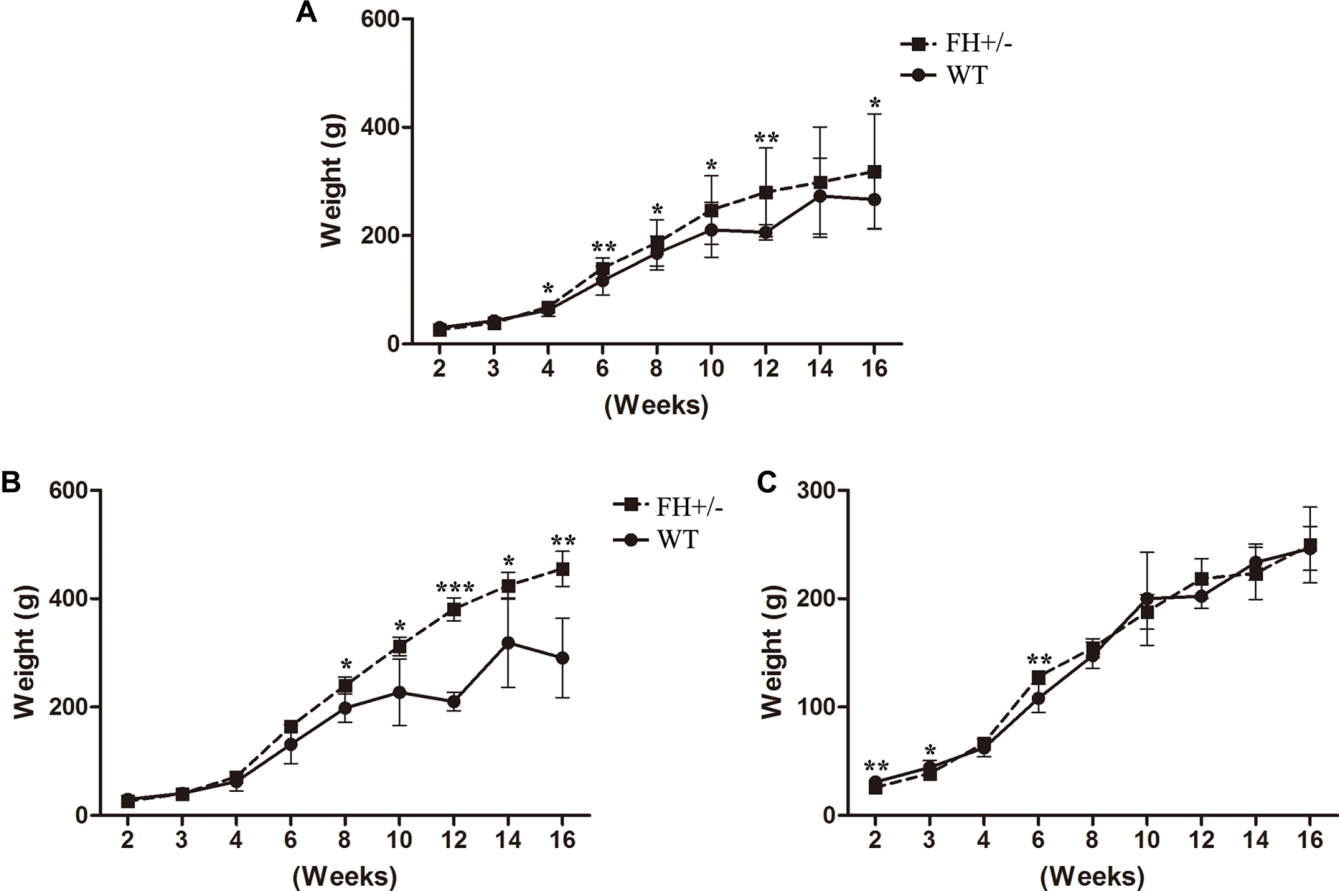
Body weight observation (**A**), (**B**) and (**C**) show the general bodyweight curves for all the experimental rats, bodyweight curves for male rats and bodyweight curves for female rats, respectively. Compared to the WT rats, the male FH+/− KO rats show significantly higher bodyweights. * means *P* < 0.05, ** means *P* < 0.01, ***means *P* < 0.001. WT: *n* = 16, male = 7, female = 9; FH+/−: *n* = 14, male = 6, female = 8.

There was no behaviour difference between the WT and FH+/− KO rats except the body weight. As shown in Figure 5A, the FH+/− male rats show significantly higher body weight within the 16-week observation period (Figure 5B), although the female FH+/− rats (Figure 5C) show no body weight difference within the same observation period. The maximum difference was observed at about 12-week in the FH+/− KO males with up to 81.0% higher body weight (*P* = 0.012).

### Clinical haematology and biochemical examinations

**Figure 6:**
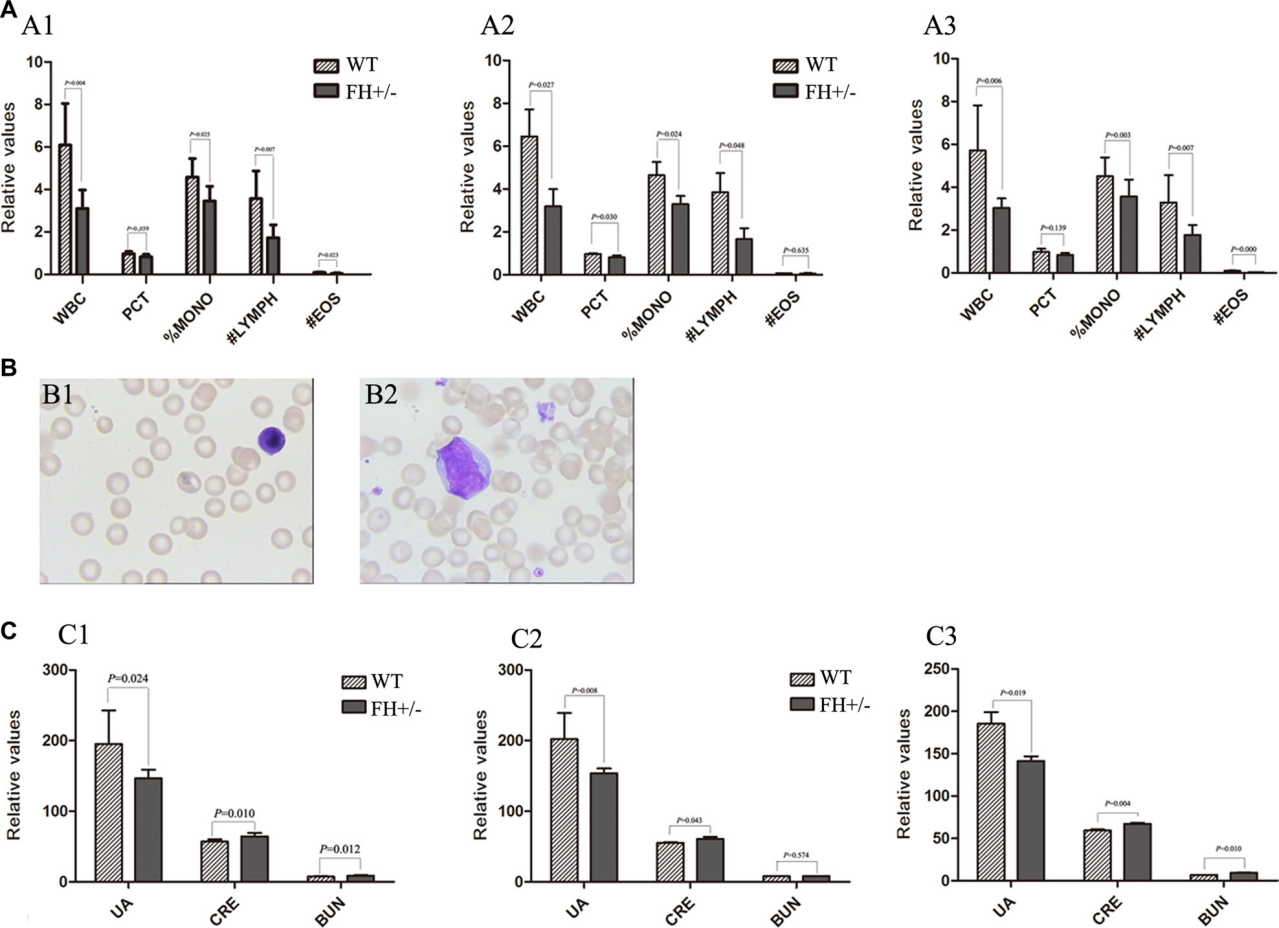
Results of haematology and biochemistry assays (**A**) shows the histograms of the blood tests of WBC, PCT, %MONO, #LYMPH and #EOS in the WT and FH+/− KO rats. The histograms of the blood tests in general, in the male and female rats are shown in A1, A2 and A3, respectively, and all show significantly decreased values of WBC, PCT, %MONO, #LYMPH and #EOS in the FH+/− KO rats. Representative lymphocyte morphology photographs are shown in (**B**). While B1 shows mature small-size lymphocyte with rather larger cytoplasm in a WT rat, B2 shows a larger lymphocyte with bizzare giant nuclear in a FH+/− KO rat. (**C**) shows the results of blood biochemistry assays in histograms. C1 shows the results in general and C2 shows the results in the males while C3 shows the results in females. All results in the experimental rats are compared to the values in the WT rats. While the UA value is significantly decreased in the FH+/− KO rats, the values of CRE and BUN in the FH+/− KO rats are significantly increased. WBC: white blood cell; %MONO: %mononucleosis; #LYMPH: #lymphocyte; #EOS: #eosimophil; UA: uric acid; CRE: creatinine; BUN: blood urea nitrogen.

To explore whether FH+/− could influence haematological and biochemical molecule changes, a series of related blood samples were examined. All the measurements are shown in Table 4. As shown in Figure 6A1, compared with the WT rats, the values of white blood cell (WBC), platelet hematocrit (PCT), %mononucleosis (%MONO), #lymphocyte (#LYMPH) and #eosimophil (#EOS) in the FH+/− KO rats were observed decreased with 49.0% (*P* = 0.004), 14.7% (*P* = 0.039), 24.5% (*P* = 0.025), 51.6% (*P* = 0.007) and 58.0% (*P* = 0.023), respectively, and the differences were consistent in both male (Figure 6A2) and female (Figure 6A3) rats. The lymphocytes of the FH+/− KO rats were found morphologically immature (Figure 6B2), compared to the lymphocytes in the control rats (Figure 6B1). In addition, it was found that blood urea nitrogen (BUN) and creatinine (CRE) in the FH+/− KO rats were increased with 20.1% (*P* = 0.012) and 12.7% (*P* = 0.010), respectively, compared to the control rats (Figure 6C1). In addition, the blood UA was decreased in the FH+/− KO rats as well, with about 25.0% reduction (*P* = 0.024), and this difference was consistent in both male (Figure 6C2) and female (Figure 6C3) rats, suggesting hematopoietic and kidney dysfunction in the FH+/− KO rats.

### Histological and immunohistochemical evaluation

**Figure 7:**
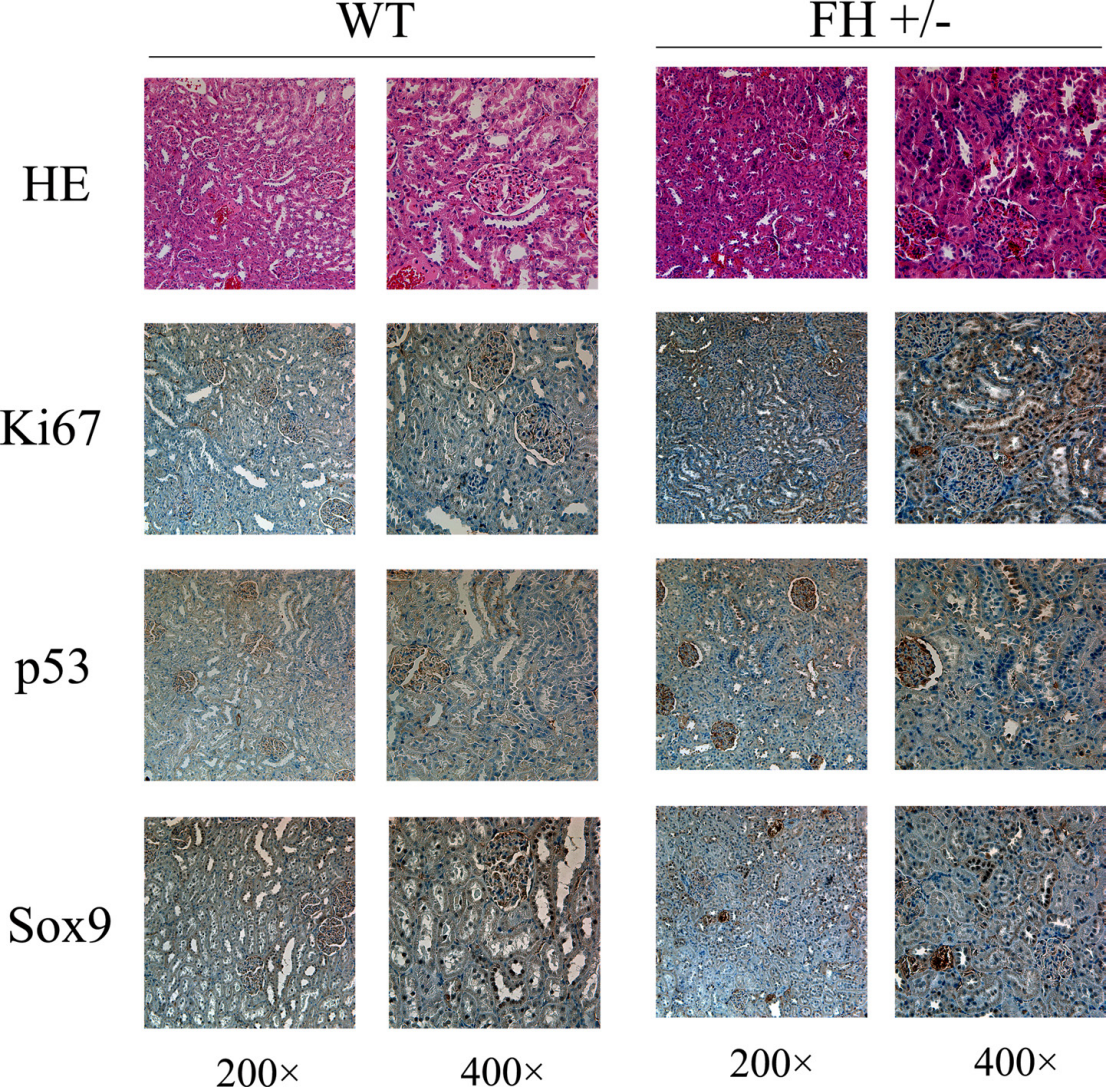
Histological and immunohistochemical results of rat kidney Histological evaluation of the haematoxylin and eosin (HE) stained slides shows variable foci of tubular epithelial cells around glomeruli with abnormal structure and the cell nuclei are prominently bigger with thicker chromatin. These abnormal tubular epithelial cells in the FH+/− KO rat kidney are more positive for the expression of Ki67, p53 and Sox9.

Based on the abnormal findings of kidney function in the FH+/− KO rats, histological and immunohistochemical evaluation of the rat kidneys was further performed (Figure 7). Histologically, small foci in the medullary part of the kidney in the FH+/− KO rats revealed anaplastic alterations with prominent pleomorphic larger nuclei and slightly coarse chromatin in the tubular epithelial cells around glomeruli. Those anaplastic larger epithelial cells were positive for Ki67, although the tubular epithelial cells in the same area in the control rats were largely negative or weakly positive. The same was for the p53 immunostaining. In the kidney tubular epithelial cells in the control rats, the p53 immunoreactivity was largely negative. However, areas of the kidney tubular epithelial cells in the same part in the FH+/− KO rats were positive for the p53 expression. More prominent difference was seen in the Sox9 immunostained slides. While the kidney tubular epithelial cells in the control rats were negative or weakly positive, the kidney tubular epithelial cells in the same part in the FH+/− KO rats were strongly positive for the Sox9 expression.

## DISCUSSION

To our knowledge, this is the first FH+/− KO rat model established using the genome editing technology TALEN. We show on this report that the mutation results in a deletion of 11 bases in the first exon in one allele and due to the non-triple base deletion, an early terminator is created in the transcript, which generates the FH+/− rats. Although it is a heterozygous knockout, decreased mRNA and protein expression of the FH could be verified in both tissues and primary cells of the FH+/− KO rats in our study. It is verified in our current study, although the new technology CRISPR/Cas9 is easier to use as indicated in literature [11–13], careful application of TALENs is still applicable in such studies. Furthermore, the established FH+/− KO rat model will be useful for the studies of energy metabolism and the Warburg effect [14] as well.

Recently, the study of the FH has been a hot issue. It has been reported that the mutation of FH gene in the NCCFH1 cell lines from a metastatic hereditary papillary renal cell carcinoma type 2 (PRCC2) results in glucose-dependent growth and impaired oxidative phosphorylation, which is consistent with the Warburg effect [9]. A clinic study shows that the mutation of FH gene results in the Reed syndrome, which is an autosomal dominant disorder characterized by cutaneous leiomyomas, uterine leiomyomas [15], and renal cell carcinoma [7, 16, 17], possibly representing genetic heritage lesions [18]. In addition, FH deficiency also could lead to metabolic disorders with severe encephalopathy, seizures and poor neurological outcome [10], suggesting an important role of FH in neurology as well. It is known that absence of FH in cells leads to an accumulation of fumarate, which will activate hypoxia-inducible factors (HIFs) at normal oxygen tensions [19, 20]. It is also known that mouse kidney cells, when their FH gene is knocked out, will survive with metabolic reprograming pathway beginning with glutamine uptake and ending with bilirubin [21]. FH-deficiency may activate the proto-oncogene ABL1 in kidney tumors, which may upregulate aerobic glycolysis via the mTOR/HIF1α pathway, being a promising target marker [22].

In our study, we have generated an FH+/− KO rat model with TALEN technology. 18 live-born targeted mutation offsprings were produced from 80 injected zygotes, with 22.5% efficiency, indicating a relatively high TALEN knockout efficiency in rat zygotes. Interestingly, in an attempt to create FH KO rat experiment with 16 pairs of FH+/− KO rats, there were no homozygous FH KO rats observed within the six-month period for a total of four generations among the offsprings, strongly implying a possibility of embryo lethality of homozygous FH KO. Indeed, the sequencing of the E8.0 and the E15.0 gestation rat embryos did not reveal any homozygous FH mutation, which strongly support the embryo lethality discovered in the mouse study by Pollard PJ et al. [19], indicating the fundamental role of this gene in the development of life. The reason that we did not observe any homozygous embryo in our current experiments may be explained by the possibility of dead embryo absorption in the early embryo development as revealed in the mouse study [19].

Furthermore, we discovered reduced reproductive capability in the FH+/− KO rats. Compared with the WT rats, the litter size was decreased when the WT rats were mated with the FH+/− KO rats, and significantly reduced when FH+/− KO rats were mated with FH+/− KO rats, with about 40.0% reduction. Collectively, these results indicate reduced quality of sperm or ovum in the FH+/− KO rats, a scientific issue worthy of further study.

Interestingly, significantly higher body weight was disclosed in the FH+/− KO male rats within the 16-week observation period, but the FH+/− KO female rats did not show such a difference, a phenomenon meriting further studies. FH is a TCA cycle enzyme localized in the mitochondrial matrix, and the loss of FH enzyme activity will lead to disrupted TCA cycle, resulting in abnormal metabolism. This may be explained for the increasing bodyweight observed in the FH+/− KO male rats, although the molecular mechanism behind this is still a matter of study. However, it is difficult to explain why the bodyweight in female FH+/− KO rats is not similarly influenced. Apparently, the role of sex hormones in such a difference should not be excluded in the future study in terms of the bodyweight influence of the FH gene knockout.

Our clinical hematology and biochemistry analysis have shown reduced WBC, PCT, %MONO, #LYMPH, #EOS and immature lymphocytes in the FH+/− KO rats. All these decreased values refer to possibly abnormal hematopoietic function. In addition, there are increased values of CRE and BUN and decreased value of UA in the FH+/− KO rats as well, an indication of kidney dysfunction as reported by Cakir M et al. [23]. Unfortunately, bone marrow materials from these rats were not available for further hematopoietic stem cell study.

FH is an essential enzyme of the TCA that catalyses the hydration of fumarate into malate. Abnormal FH function/expression impairs mitochondrial function directly and has indirect effects on both glucose metabolism and oxidative phosphorylation by inappropriate activation of HIF1α and NRF2 [24, 25]. Recently, there are reports showing that the mutation of FH may initiate renal cell carcinogenesis [7, 9, 18, 26–29], implying tumor suppressor function of this gene. In our current study, we have histologically and immunohistochemically examined the kidney tissues. Histological evaluation of kidney samples does reveal foci of tubular epithelial cells around glomeruli in the FH+/− KO rats with anaplastic alterations, and these cells express higher levels of Ki67, p53 and Sox9. These findings may explain the kidney dysfunction observed by the blood biochemical examinations. Although we did not observe tumor formation in the kidneys of the FH+/− KO rats, the anaplastic lesions in the medullary field of the kidney may indicate an early tumor development process, and this finding merits additional studies. Ki67 is a cellular proliferationmarker and its high expression is always associated proliferation activity of tumor cells [29]. p53 is a well-known tumor suppressor gene [30], and its high expression is often linked to p53 mutation, widely discovered in tumors. In addition, Sox9 is defined as a specific cancer stemness biomarker [31]. All these findings highlight the possibility of premalignant lesions of the epitheleial cells around the glomeruli in the FH+/− KO rats, although the p53 gene activity in these rats is current not clear.

In summary, we have successfully generated a TALEN-mediated FH+/− knockout rat model, and shown that homozygous FH knockout is embryonically lethal. The FH+/− KO leads to reduced gene and protein expression of FH in both tissues and primary cells, and the FH+/− KO rats show decreased litter size and increased body weight in male rats. In addition, the FH+/− KO rats show variable hematopoietic and kidney dysfunction revealed by blood examinations. Collectively, our results indicate the potential value of this FH+/− KO rat model in the studies of metabolic disorders and tumorigenesis *in vivo*.

## MATERIALS AND METHODS

### Animals

The study was approved by our Animal Research Committee (notion no. SYXK 2011-0001) and performed at the Laboratory of Experimental Animal Center of Henan Province in accordance with Institutional guidelines. Eight female and eight male Sprague-Dawley (SD) rats, initially average body weight of 220 g, were used in this study, and all efforts were made to minimize distress. All the animals were kept in cages quilted with shavings and at a controlled temperature of (24 ± 2)°C under a 12 h light–dark cycle with free access to water and feed for 6 months. All animals received humane care in compliance with the ethical standards.

### TALEN construction

A pair of TALENs targeting exon 1 of the FH gene was selected as a target by using a web-based tool called TAL Effector-Nucleotide Targeter (https://tale-nt.cac.cornell.edu/) [32]. Fok I nuclease functions as a dimer when it plays a key role to make double-strand breaks (DSB) in the DNA sequences, especially the length of the spacer can significantly affect the specificity of the TALENs. Therefore the TALENs were designed in pairs to precisely bind opposing DNA target sequence, which were separated by the spacer, in order to search for the potential targets. The optimal length for the activity of TALEN should be between 14-20bp and the length of the repeat units should be 16-18bp. Moreover, the TALEN target sequence was chosen in such a principle that each target sequence started after a T base and ended with a T base, which is illustrated in Figure 1A. TALENs were assembled using the TALE Toolkit (Viewsolid biotech, China) according to the manufacturer's introduction. Final constructs were produced in the pCMV-T7-tale-FokI-L/R backbone plasmid. Four pairs of TALEN plasmids were designed as shown in Figure 1B and the targeted sequences are shown in Table 1. TALEN mRNA was recovered by LiC1, washed and then resuspended in DEPC-treated H2O. The plasmids were extracted using E.Z.N.A^®^ Plasmid Midi Kit (OMEGA, USA), according to the manufacturer›s instructions. The most effective plasmid was screened by the Luciferase SSA kit (Viewsolid biotech, China). TALEN mRNA was stored at −80°C until used for embryo injection.

### Embryo microinjection

Female embryo donors were superovlated with 25IU of pregnant mare serum gonadotropin (Millipore, USA), followed by 25 IU of human chorionic gonadotropin (Millipore, USA) after 24 h, then caged with a male rat individually. The following day, donors were sacrificed and embryos were collected from the oviducts and incubated in M16 (Millipore, USA) at 37°C in 5% CO_2_ air. Fertilized one-cell embryos were transferred to medium for microinjection and the mRNAs of the pair of most effective TALEN plasmids were mixed and injected into the cytoplasm. Embryos that survived the injection procedure were surgically transferred to the oviduct of SD female rats.

### Mutation analysis

Offsprings from injected embryos were obtained and verified for the mutations of FH gene. Briefly, the tail snips (~1 cm) were cut from the 2-weeks age of offspring rats and the DNA were extracted by using the Tissue DNA Kit (OMEGA, USA) following the manufacturer's introduction. Then the extracted DNA was amplified by PCR, and the primers are shown in Table 2. The conditions for PCR analyses were as following: 98°C 3 minutes; 98°C 10s, 60°C 20s, 72°C 20s, 35 cycles; 72°C 2 minutes and 4°C preservation. Subsequently, the products of PCR were sent to a company (Sagon biotech, China) to verify the mutation.

**Table 2:** Primers for sequencing

gene name		sequence (5′ to 3′)
FH	sense strand	F- CTGGGCAGTATGTGAATTGTATAAAC
	antisense strand	R- GAACCCTGACTAAAACAGCCC

### Biological behaviour analysis

Offspring of the verified FH+/− KO rats were mated with FH+/− KO or with wild-type (WT) rats, and the birth rate was recorded, with the wild type male/female or female/male mating SD rats as control. Meantime, the clinical observation was recorded including body weight, fur coloration and life behaviour. The bodyweight of the paired WT and FH+/− KO SD rats that were born at the same time was examined every two weeks. In order to explore whether there were homozygous FH KO embryos, the pregnant potential KO female rats were sacrificed at E8.0 or E15.0, and the embryos were applied for DNA sequences as mentioned above.

### Primary cells extraction

The experimental rats were euthanized with 10% chloral hydrate and the lungs were collected as soon as possible. The materials were placed in PBS at 4°C, washed with new PBS and cut to tiny pieces before incubated with trypsin at 37°C 5 minutes as reported [33, 34]. Then tissue fragments inside the digestion solution were pipetted up and down to break the clumps and the supernatant solution was transferred to sterile 50 mL tubes containing warm DMEM/F12 media with 15% FBS and 1X antibiotic/antimycotic solution. The above procedures were repeated 8–10 times, and all the solution containing singular cells was collected and centrifuged for 5 minutes at 1,000 g. The cells were then placed into a 10 cm culture dish with 10 ml warm DMEM/F12 media with 15% FBS, 1× antibiotic/antimycotic solution and incubated for 2h at 37°C in 5%CO_2_ before the medium was replaced with new one.

### Real-time quantitative polymerase chain reaction (RT-qPCR) Analysis

Total RNAs were isolated from tissue samples of rats using the Trizol (Invitrogen, USA) with the glass homogenizer at room temperature (RT). RNA concentration was determined by spectrophotometric analysis (Eppendorf, Germany), and the integrity was tested by the OD260nm/OD280nm absorption ratio (>1.7,<2.0). Reverse transcription reaction was applied with 2 μg of total RNA using the RevertAid First Strand cDNA Synthesis kit (Thermo Scientific, USA) according to the manufacturer's instruction. Amplification reaction was performed by using the MiniOpticon detection system (Bio-Rad, USA) with the SYBR Premix Ex Taq II (Takara, Japan). The primers are shown in Table 3 and all PCRs were performed in duplicate and the reaction conditions are as following: 30s at 95°C, 40 cycles of 5s at 95°C and 30s at 60°C (LightCycler480, Roche, USA). The results of FH mRNA expression levels were calculated by using the comparative cycle threshold (CT) and the relative changes in the FH level were analysed with the 2^−ΔΔCT^ formula.

**Table 3:** Primers for RT-qPCR

gene name		sequence (5′ to 3′)
FH	sense strand	F-CTGGGCAGTATGTGAATTGTATAAAC
	antisense strand	R-GAACCCTGACTAAAACAGCCC

**Table 4:** Results of hematology, blood chemistry

Test	WT			KO		
	Total (*n* = 6)	Male (*n* = 3)	female (*n* = 3)	Tota (*n* = 6)	Male (*n* = 3)	female (*n* = 3)
Hematology						
White blood cells (×10^9^/L)	6.1 ± 1.2	6.5 ± 1.4	5.7 ± 1.1	3.1 ± 0.6**	3.2 ± 0.9*	3.0 ± 0.4**
Red blood cells (×10^12^/L)	7.9 ± 0.9	8.6 ± 0.2	7.2 ± 0.8	8.5 ± 0.5	8.9 ± 0.3	8.1 ± 0.3
Haemoglobin (g/L)	139.0 ± 11.7	146.0 ± 5.0	132.0 ± 13.1	143.9 ± 6.0	145.0 ± 7	145.0 ± 7
Hematocrit (%)	41.7 ± 3.0	43.4 ± 1.6	39.9 ± 3.3	41.9 ± 1.5	42.1 ± 1.6	41.7 ± 1.6
Platelets (×10^9^/L)	1129.3 ± 150.4	1106.3 ± 112.2	1152.3 ± 205.8	981.3 ± 113.0	1004.0 ± 139.6	964.3 ± 107.8
Mean platelet volume (%)	8.6 ± 0.2	8.7 ± 0.3	8.5 ± 0.2	8.4 ± 0.5	8.1 ± 0.5	8.7 ± 0.4
Platelet hematocrit (fL)	1.0 ± 0.1	1.0 ± 0.1	1.0 ± 0.2	0.8 ± 0.1*	0.8 ± 0.1*	0.8 ± 0.1
Mean corpusular volume (fL)	52.9 ± 3.6	50.3 ± 0.9	55.6 ± 3.4	49.6 ± 2.5	47.2 ± 1.4	51.4 ± 1.1
Mean corpusular hemoglobin (pg)	17.6 ± 1.0	16.9 ± 0.3	18.3 ± 0.8	17.1 ± 0.9	16.2 ± 0.5	17.7 ± 0.4
Mean corpusular hemoglobin	333.5 ± 5.8	336.7 ± 0.6	330.3 ± 7.3	343.7 ± 2.9	344.3 ± 4.1	343.3 ± 2.2
concerntration (g/L)						
Neutral (%)	Not detected	Not detected	Not detected	Not detected	Not detected	Not detected
Lymphocyte (%)	57.8 ± 6.3	58.5 ± 5.9	57.1 ± 7.9	54.8 ± 7.8	50.7 ± 7.0	57.8 ± 7.9
Mononucleosis (%)	4.6 ± 0.6	4.6 ± 0.1	4.5 ± 0.9	3.4 ± 0.7*	3.3 ± 0.7*	3.6 ± 0.8**
Eosimophil (%)	1.6 ± 0.8	1.0 ± 0.2	2.1 ± 0.9	1.0 ± 0.8	1.0 ± 0.9	1.0 ± 0.1
Basophilic leukemia (%)	Not detected	Not detected	Not detected	Not detected	Not detected	Not detected
Neutrophil (#)	Not detected	Not detected	Not detected	Not detected	Not detected	Not detected
Lymphocyte absolute value (#)	3.6 ± 1.1	3.8 ± 1.0	3.3 ± 1.3	1.7 ± 0.6**	1.7 ± 0.9*	1.8 ± 0.5**
Mononucleosis (#)	0.3 ± 0.1	0.3 ± 0.2	0.3 ± 0.1	0.2 ± 0.3	0.4 ± 0.5	0.1 ± 0.0
Eosimophil (#)	0.09 ± 0.03	0.06 ± 0.01	0.1 ± 0.0	0.04 ± 0.04*	0.04 ± 0.06	0.03 ± 0.00***
Basophilic leukemia (#)	Not detected	Not detected	Not detected	Not detected	Not detected	Not detected
Red blood cell volume distribution width standard deviation (%)	25.8 ± 1.9	25.5 ± 0.5	26.1 ± 2.9	26.8 ± 1.7	28.0 ± 1.7	25.6 ± 1.2
Red blood cell volume distribution width coefficient of variation (%)	16.0 ± 2.6	17.3 ± 0.7	14.6 ± 3.3	17.5 ± 2.3	19.6 ± 1.4	16.0 ± 1.2
Platelet distribution width (fL)	9.6 ± 0.6	9.8 ± 0.7	9.5 ± 0.5	9.4 ± 0.9	8.9 ± 1.0	9.8 ± 0.7
Platelet larger cell ratio (%)	15.7 ± 2.0	16.5 ± 2.5	14.8 ± 1.5	14.3 ± 3.8	11.7 ± 3.8	16.1 ± 2.8
Blood chemistry variables						
Alanine amio transferase (IU/L)	41.8 ± 6.6	47.3 ± 3.1	36.3 ± 3.2	30.4 ± 19.2	43.7 ± 5.7	35.8 ± 11.5
Aspartate amino transferase (IU/L)	122.5 ± 14.0	130.3 ± 10.1	114.7 ± 14.4	86.1 ± 50.6	124.3 ± 9.7	100.5 ± 9.8
γ-Glutamyl transpeptadase (IU/L)	1.2 ± 0.4	1.3 ± 0.6	1.0 ± 0.0	1.2 ± 0.8	1.3 ± 0.6	1.8 ± 0.5
Alkaline phosphatase (IU/L)	60.3 ± 34.1	84.7 ± 31.0	36.0 ± 13.1	40.0 ± 27.6	69.3 ± 4.5	38.0 ± 9.4
Total bilirubin (umol/L)	−0.2 ± 0.1	−0.2 ± 0.1	−0.2 ± 0.1	−0.03 ± 0.36	−0.3 ± 0.3	0.2 ± 0.4
Direct bilirubin (umol/L)	0.3 ± 0.1	0.2 ± 0.1	0.4 ± 0.2	0.2 ± 0.2	0.3 ± 0.2	0.4 ± 0.2
Indirect bilirubin (umol/L)	0.3 ± 0.2	0.4 ± 0.1	0.1 ± 0.2	−0.3 ± 0.3	−0.6 ± 0.2	−0.2 ± 0.3
Total protein (g/L)	64.8 ± 6.4	64.4 ± 1.6	65.1 ± 9.9	51.4 ± 29.3	64.0 ± 1.3	67.7 ± 2.9
Albumin (g/L)	34.5 ± 3.0	33.5 ± 1.7	35.5 ± 4.1	27.9 ± 15.9	33.9 ± 0.9	37.3 ± 2.2
Globulin (g/L)	30.3 ± 3.9	31.0 ± 0.2	29.6 ± 6.0	23.6 ± 13.4	30.1 ± 1.1	30.5 ± 0.8
Glucose (mmol/L)	7.3 ± 1.3	8.2 ± 0.3	6.4 ± 1.4	5.1 ± 3.0	7.2 ± 0.4	6.0 ± 1.2
Blood urea nitrogen (mmol/L)	7.3 ± 0.7	7.8 ± 0.5	6.7 ± 0.3	8.7 ± 1.0*	8.1 ± 0.5	9.2 ± 1.0*
Uric acid (umol/L)	162.8 ± 81.0	202.0 ± 12.5	123.7 ± 107.9	146.6 ± 12.3*	153.7 ± 11.9**	141.3 ± 10.9*
Serum creatinine (IU/L)	886.2 ± 413.7	1094.3 ± 524.4	678.0 ± 150.9	1014.7 ± 581.6*	1238.7 ± 170.2*	846.8 ± 754.6**
Creatine kinase isoenzymes-MB (IU/L)	35.8 ± 5.2	36.7 ± 7.6	35.0 ± 2.6	37.3 ± 5.3	41.0 ± 1.0	34.5 ± 5.6
Creatinine (umol/L)	57.2 ± 2.9	55.5 ± 1.7	59.3 ± 2.1	64.4 ± 4.2	60.7 ± 2.9	67.3 ± 2.1

Where appropriate, data are presented as the mean ± the standard deviation.

* *P* < 0.05 (paired *t*-test) vs WT,

** *P* < 0.01 (paired *t*-test) vs WT,

*** *P* < 0.001 (paired *t*-test) vs WT.

### Western blotting

After the rats were euthanized, separate tissues of heart, liver, lung, kidney, brain, spleen, stomach and testis were collected as soon as possible and then placed in PBS at 4°C before washed and grinded in ice-cold RIPA lysis buffer by using the glass homogenizer. The lysates were then centrifuged and the supernatants were assayed for protein content. About 50 μg of proteins were fractionated on 10% polyacrylamide gel and transferred onto PVDF membrane from Millipore Corporation (Billerica, MA, USA). 50mM Tris-HCl (pH 7.5) buffer, 150 mM NaCl and 0.1% Tween 20 ((TBS-T)) containing 5% non-fat dry milk to block the nonspecific binding sites of the membrane for 1 h at RT and incubated with primary anti-FH antibody (Abcam, USA) diluted in blocking buffer overnight at 4°C. Membranes were incubated with the respective horseradish peroxidase-labelled secondary antibody for 1 h at RT. Then the proteins were revealed using a Pierce ECL kit (Thermo Scientific, USA) following the manufacturer's instructions and visualized by ChemiDoc XRS^+^ system (Bio-rad, USA).

### Immunofluorescence microscopy

The primary fibroblast cells were obtained from the newborn rats as described above and plated on poly-L-lysine coated coverslip, washed with PBS twice, fixed in 4% paraformaldehyde in PBS for 30 minutes and permeabilized in 0.1% Triton X-100 in PBS for 5 minutes at RT. Nonspecific binding sites were blocked with 5% BSA in PBS (incubation buffer) for 30 minutes and the primary FH antibody was incubated overnight at 4°C. On the following day after extensive washings with PBS, the corresponding secondary FITC-labelled antibody was added and incubated with the cells for 1 h. Cells were then washed and stained with Hoechst33342 for nuclear visualisation. Confocal images were then obtained using the microscope Olympus FV1200 (Olympus, Japan).

### Laboratory blood examinations

Rats were anesthetized with 10% chloral hydrate after having fasted overnight and the blood samples were obtained from the abdominal aorta before euthanized. Blood samples were collected with uncoated, EDTA and citrate-coated tubes, and the hematologic parameters were analysed with Siemens Advia 2120 (Siemens, USA) for the following cuntings/measurements: white blood cell (WBC), red blood cell (RBC), haemoglobin (HGB), hematocrit (HCT), platelet count (PLT), mean platelet volume (MPV), platelet hematocrit (PCT), mean corpusular volume (MCV), mean corpusular hemoglobin (MCH), mean corpusular hemoglobin concerntration (MCHC), %neutral (%NEUT), %lymphocyte (%LYMPH), %mononucleosis (%MONO), %eosimophil (%EOS), %basophilic leukemia (%BASO), #neutrophil (#NEUT), #lymphocyte (#LYMPH), #mononucleosis (#MONO), #eosimophil (#EOS), #basophilic leukemia (#BASO), red blood cell volume distribution width standard deviation (RDW-SD), red blood cell volume distribution width coefficient of variation (RDW-CV), platelet distribution width (PDW) and platelet larger cell ratio (P-LCR). Additional, blood tests were performed with a Synchron Cx 5 clinical system Beckman Coulter (USA) for the followings: alanine amio transferase (ALT), aspartate amino transferase (AST), γ-glutamyl transpeptadase (GGT), alkaline phosphatase (ALP), total bilirubin (TBIL), direct bilirubin (DBIL), indirect bilirubin (IBIL), total protein (TP), albumin (ALB), globulin (GLB), glucose (GLU), blood urea nitrogen (BUN), uric acid (UA), creatinine (CRE), cardiac Marker (CK) and creatine kinase isoenzymes (CK-MB). Furthermore, blood smears were prepared and stained with the Wright method in order to detect the morphology of the cells.

### Histological evaluation

Rats were euthanized with chloral hydrate at the end of the experiment and the kidneys were collected for histological sample preparation. Formalin-fixed paraffin-embedded tissue sections were cut and stained with Harris hematoxylin (Sigma-Aldrich, USA) and eosin solution (Sigma-Aldrich, USA). Briefly, slides were deparaffinized in deionized water and then stained with hematoxylin for 3 minutes, rinsed under running tap water, 70 % ethanol and then stained with eosin for 3 minutes before the slides were rinsed and dehydrated in ethanol, cleared in xylene, and then mounted by machine (Dako, Denmark). The slides were histologically evaluated under an optical double-headed microscope (Olympus, Tokyo, Japan).

### Immunohistochemistry (IHC)

The paraffin-embedded tissues were cut to 3-μm sections. Dako Envision FLEX+ system (K8012; Dako, Glostrup, Denmark) was used to deparaffinized and epitopes were unmasked in PT link with low pH target retrieval solution (Dako, Denmark). Briefly, the slides were blocked for 5 minutes with peroxidase blocking solution (Dako) at room temperature (RT), and then incubated with rabbit polyclonal Ki67 antibody (cat. no. ab15580; 1:800; Abcam, USA), rabbit polyclonal p53 antibody (cat. no. ab131442; 1:500; Abcam, USA) and rabbit polyclonal sox9 antibody (cat. no. ab26414; 1:1000; Abcam, USA) at 4°C overnight before incubated with rabbit linker (Dako, Denmark) for 15 minutes, horseradish peroxidase (Dako) for 30 minutes at RT. The slides were subsequently stained with 3,3′-diaminobenzidine tetrahydrochloride for 10 minutes, counter-stained with hematoxylin, dehydrated, and mounted in Richard-Allan Scientific Cytoseal XYL (Thermo Fisher Scientific, Waltham, MA, USA) before evaluated under an optical double-headed microscope (Olympus, Tokyo, Japan).

### Statistics

All values are expressed as mean ± SD, and the student *t-test* was used to evaluate the significance differences between the two groups. The significance level was set as *P* < 0.05.
